# Patients Undergoing Surgery for Hip Fractures Suffer from Severe Oxidative Stress as Compared to Patients with Hip Osteoarthritis Undergoing Total Hip Arthroplasty

**DOI:** 10.1155/2021/5542634

**Published:** 2021-06-09

**Authors:** Theofilos Karachalios, Dionyssios Paridis, Fotios Tekos, Zoi Skaperda, Aristidis S. Veskoukis, Demetrios Kouretas

**Affiliations:** ^1^Department of Orthopaedics and Musculoskeletal Trauma, University General Hospital of Larissa, School of Health Sciences, Faculty of Medicine, University of Thessaly, Viopolis, Mezourlo Region, Larissa 41500, Greece; ^2^Department of Biochemistry and Biotechnology, University of Thessaly, Viopolis, Mezourlo region, 41500 Larissa, Greece; ^3^Department of Nutrition and Dietetics, University of Thessaly, Argonafton 1, 42132 Trikala, Greece

## Abstract

Hip fractures are associated with the highest degree of morbidity and mortality of all fractures in elderly patients and pose a major risk for subsequent fractures. Patients with hip fractures also present accelerated bone turnover despite early stable fracture fixation and early mobilization. We aimed to evaluate oxidative stress in two groups of patients (25 patients each, matched for age, side, and BMI) who underwent internal fixation of hip fractures and total hip arthroplasty for hip osteoarthritis. Blood samples were taken from all patients during admission, the day of surgery, the 4^th^ postoperative day, and the 15^th^ postoperative day. Reduced (GSH) and oxidized (GSSG) glutathione, GSH/GSSG, catalase (CAT), thiobarbituric acid reactive substances (TBARS), protein carbonyls (PC), and total antioxidant capacity (TAC) as a widely used battery of redox biomarkers were recorded from blood samples. Patients with hip fractures who undergo fixation surgery, compared to those with hip osteoarthritis, suffer significant oxidative stress with an active but insufficient first line of oxidative defense, an intensive first line reaction, a very active second line of oxidative defense, and a low plasma antioxidant capacity. Surgery worsened already present lipid- and protein-related tissue damage. The severe oxidative stress observed may explain high morbidity and mortality rates and high bone turnover status, as well as the high incidence of refractures. Furthermore, the question of whether antioxidant therapy measures should be introduced in the management of hip fracture patients is raised.

## 1. Introduction

Musculoskeletal system trauma and pathology in a globally ageing population cause morbidity, mortality, and high health system expenditure [1]. Despite major progress having been made in prevention, diagnosis, and medical and surgical treatment, osteoporosis, sarcopenia, falls, and fractures are still major health concerns [2]. Hip fractures show a high incidence of postoperative complications, low levels of functional recovery, and high morbidity and mortality rates [3–6]. The quality of life of these patients is also negatively affected by morbidity and postoperative complication rates [7, 8]. As a result, hip fractures have come to the attention of both health system professionals and economic providers [4, 6–8].

In women, fast bone loss starts with the menopause and is followed, some years later, by slower, lifelong, and age-related bone loss [9–11]. Fast bone loss is mainly due to decreased sex hormone levels, while age-related slow bone loss is due to a still present hormone deficiency, changes in the levels of oxidative stress, and/or age-related proinflammatory status [12, 13]. At a cellular level, bone loss in osteoporosis is a result of imbalanced (high turnover status) bone remodeling in which bone resorption is not compensated by bone formation [9–11]. However, the biological effects of this negative remodeling process do not fully explain the high rate of postoperative complications, morbidity, and mortality seen in older patients with hip fractures [14].

We have previously shown that patients who have undergone hip surgery (total hip arthroplasty) temporarily become fast bone losers for at least 6 months postsurgery [15]. We have also shown that older patients who have sustained a hip fracture and have undergone internal fixation become super-fast bone losers for at least a period of 1 year [16, 17]. The exact cause of this state of high bone turnover remains unknown, and other metabolic pathways and possible treatments should be explored.

The working hypothesis of this study is that patients who sustain hip fractures and undergo surgery, in contrast to patients who undergo hip surgery for osteoarthritis, reach a metabolic status of excessive oxidative stress which is responsible for excessive bone loss, slow functional recovery, and high rates of morbidity and mortality. This comparison will allow us to draw conclusions as to whether hip fracture and/or hip surgery are responsible for excessive oxidative stress response. We present comparative data, related to oxidative stress factors/redox biomarkers, between two groups of patients who underwent internal fixation of hip fractures (Group A) and total hip arthroplasty for hip osteoarthritis (Group B).

## 2. Materials and Methods

### 2.1. Patients

Twenty-five consecutive female patients (Group A) aged between 70 and 80 years who had sustained a hip fracture and had been admitted to our Orthopaedic Department for surgery were included in the study. Another 25 female patients, matched for age, body mass index, and side, who had also been admitted to our department during the same period of time for hip osteoarthritis and had undergone arthroplasty surgery, were included in the study as the control group (Group B). Patients who had a history of bone metabolic disease and who were taking medications affecting bone metabolism and/or taking medications and dietary supplements affecting redox balance were excluded from the study. In more detail, subjects suffering from autoimmune diseases (e.g., rheumatoid arthritis), endocrinic disorders, dementia, severe comorbidities of other organ systems, or using antiosteoporotic treatment or other agents which affect bone metabolism or having a history of previous vertebral or hip fracture were excluded from the study. Additionally, subjects who had undergone recent surgery or a delay of the hip operation for more than 48 hours and/or the need for postoperative restriction of movement were excluded from the study. Nonambulatory patients, alcohol abusers, and smokers were also excluded. Measurements of the levels of 25-vitamin D3, intact parathyroid hormone (PTH), and thyroid stimulating hormone (TSH) were carried out in order to exclude serious endocrine disorders that might affect bone metabolism. Written informed consent forms were obtained from all patients, and the study was approved by the National Ethical Committee.

Blood samples were taken from all patients during admission, the day of surgery, the 4^th^ postoperative day, and the 15^th^ postoperative day. The following redox biomarkers were measured: reduced (GSH) and oxidized form (GSSG) of glutathione as the most potent intrinsic antioxidant molecule, the GSH/GSSG ratio, catalase (CAT) as a fundamental antioxidant enzyme, thiobarbituric acid reactive substances (TBARS) as a biomarker of lipid peroxidation, protein carbonyls (PC) as a biomarker of protein oxidation, and total antioxidant capacity (TAC) as a crude biomarker for assessing blood antioxidant potency [18, 19].

### 2.2. Blood Sampling

Five mL of venous blood was collected from each patient. One mL of whole blood was transferred into a tube containing dipotassium ethylenediamine tetra-acetic acid (K2EDTA) as an anticoagulant agent. A solution of 5% trichloroacetic acid (TCA) was added to the whole blood (1 : 1 *v*/*v*) for the precipitation of the erythrocyte membranes after centrifugation (4,000 × g, 10 min, 4°C). The supernatant was removed, added to 5% TCA (3 : 1 *v*/*v*), and centrifuged (28,600 × g, 5 min, 4°C). The last step was repeated twice. Finally, the whole blood lysate was collected and used for the measurement of the reduced (GSH) and oxidized (GSSG) glutathione. Four mL of whole blood was transferred into a tube containing K2EDTA and left at room temperature to coagulate. The tube was then centrifuged (1,500 × g, 10 min, 4°C). Serum was separated into aliquots and used for the determination of CAT, TBARS, PC, and TAC.

### 2.3. Chemicals

Hydrogen peroxide (H_2_O_2_) was purchased from Merck (Darmstadt, Germany). 5,5′-Dithiobis(2-nitrobenzoic acid) (DTNB), 4-dinitrophenylhydrazine (DNPH) and 1,1-diphenyl-2-picrylhydrazyl (DPPH) were obtained from Sigma-Aldrich (St. Louis, MO, USA) while all other chemicals and solvents used in this study were of the highest purity commercially available.

### 2.4. Protocols for the Measurement of Redox Biomarkers

#### 2.4.1. GSH and GSSG

GSH and GSSG were measured according to Reddy et al. [20] and Tietze [21], as previously described [22]. For GSH, 20 *μ*L of whole blood treated with TCA was mixed with 660 *μ*L of 67 mM sodium potassium phosphate (pH 8.0) and 330 *μ*L of DTNB. The samples were incubated in the dark at room temperature (RT) for 45 min, and the absorbance was monitored at 412 nm. GSSG was assayed by treating 260 *μ*L of whole blood with TCA, to be neutralized up to pH 7.0–7.5 with NaOH. Four microliters of 2-vinyl pyridine were added, and the samples were incubated for 2 h at RT. Five microliters of whole blood treated with TCA were mixed with 600 *μ*L of 143 mM sodium phosphate (6.3 mM EDTA, pH 7.5), 100 *μ*L of 3 mM NADPH, 100 *μ*L of 10 mM DTNB, and 194 *μ*L of distilled water. The samples were incubated for 10 min at RT. After the addition of 1 *μ*L of glutathione reductase, the change in absorbance at 412 nm was monitored for 3 min.

#### 2.4.2. Catalase

Catalase is a crucial antioxidant enzyme used for the reduction of H_2_O_2_ to H_2_O and O_2_. According to Aebi's method, 2975 *μ*L of 67 mM sodium potassium phosphate (pH 7.4) was added to 20 *μ*L of serum, and the samples were incubated at 37°C for 10 min [23]. Five microliters of 30% H_2_O_2_ were added to the samples, and the change in absorbance was immediately monitored at 240 nm for 1.5 min.

#### 2.4.3. Thiobarbituric Acid Reactive Substances

The measurement of TBARS is a well-established method for monitoring lipid peroxidation [24]. Decomposition of the unstable peroxides derived from polyunsaturated fatty acids results in the formation of malondialdehyde (MDA), which can be quantified following its controlled reaction with thiobarbituric acid (TBA). TBARS are expressed in terms of MDA equivalents. MDA forms a 1 : 2 adduct with TBA, which can be measured spectrophotometrically. A mixture of 100 *μ*L of serum with 500 *μ*L of TCA 35% and 500 *μ*L of Tris-HCl (200 mM, pH 7.4) was incubated for 10 min at RT. One milliliter of 2 M Na_2_SO4 and 55 mM thiobarbituric acid solution was added, and the samples were incubated at 95°C for 45 min. The samples were cooled on ice for 5 min and were vortexed after adding 1 mL of TCA 70%. The samples were centrifuged (15,000 g, 3 min), and the absorbance of the supernatant was monitored at 530 nm.

#### 2.4.4. Protein Carbonyls

Protein carbonyls are one of the generic and most reliable biomarkers of protein oxidation and the most frequently utilized. Carbonyl groups, namely, aldehydes and ketones, are produced on a protein side chain of specific amino acids like proline, arginine, lysine, and threonine. They are preferred markers because of the fact that they are stable moieties. Carbonyl formation was detected by a reaction with DNPH and its conversion to 2,4-dinitrophenylhydrazone (DNP-hydrazone) [25]. This reaction was measured spectrophotometrically. The assay was carried out by adding 50 *μ*L of 20% TCA to 50 *μ*L of serum, and this mixture was incubated in an ice bath for 15 min and centrifuged (15,000 g, 5 min, 4°C). The supernatant was discarded, and 500 *μ*L of 10 mM 2,4-dinitrophenylhydrazine (in 2.5 N HCL) for the sample, or 500 *μ*L of 2.5 N HCL for the blank, was added to the pellet. The samples were incubated in the dark at RT for 1 h, with intermittent vortexing every 15 min, and were centrifuged (15,000 g, 5 min, 4°C). The supernatant was discarded, and 1 mL of 10% TCA was added, vortexed, and centrifuged (15,000 g, 5 min, 4°C). The supernatant was discarded, and 1 mL of ethanol-ethyl acetate (1 : 1 *v*/*v*) was added, vortexed, and centrifuged (15,000 g, 5 min, 4°C). The washing step was repeated twice. The supernatant was discarded, and 1 mL of 5 M urea (pH 2.3) was added, vortexed, and incubated at 37°C for 15 min. The samples were centrifuged (15,000 g, 5 min, 4°C), and the absorbance was monitored at 375 nm. Total serum protein was measured using a Bradford reagent from Sigma Chemical Co. (St. Louis, MO, USA).

#### 2.4.5. Total Antioxidant Capacity

Total antioxidant capacity (TAC) of serum is evaluated using DPPH^·^ [26]. In the presence of a hydrogen donor existing in the serum, the free radical DPPH^·^ is reduced to the 1,1-diphenyl-2-picrylhydrazine. The depletion of the radical is evaluated spectrophotometrically by the decrease of the absorbance at 520 nm. The reaction was carried out by adding 20 *μ*L of serum in 480 *μ*L of 10 mM sodium potassium phosphate (pH 7.4) and 500 *μ*L of 0.1 mM DPPH^·^. The samples were incubated in the dark for 30 min at RT and centrifuged (20,000 g, 3 min), and the absorbance was monitored at 520 nm.

### 2.5. Statistical Analysis

*ΑΝΟ*V*Α* for repeated measurements and the Mann-Whitney *U* test were used in order to develop differences within and between groups. The level of statistical significance was set at *p* < 0.05. Data are presented as mean ± SEM.

## 3. Results

All patients, in both groups, were operated within 48 h of admission and were mobilized the first postoperative day. Patients in Group A had an average BMI of 24.9 (range 20.1 to 27.2) and in Group B an average of 25.2 (range 19.8 to 26.8). Patients in both groups had ordinary pre- and postoperative diets with no effect on metabolism. Three patients in Group B and two in Group A stated that they were past but currently not active smokers (discontinuation of smoking of more than 5 years). No patient was lost from follow-up.

### 3.1. GSH and GSSG

**Figure 1:**
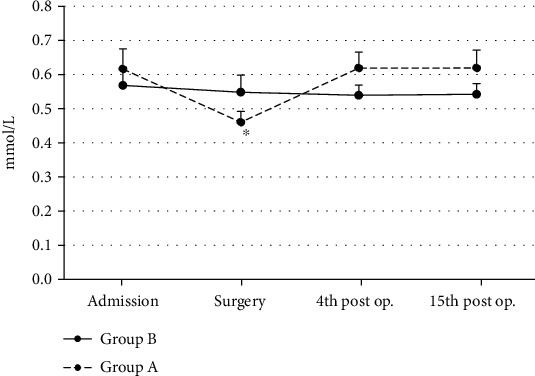
Reduced glutathione (GSH) mean values in both groups (*n* = 25 for each group) are shown. ^∗^Significantly different (*p* < 0.05) within groups compared with hospital entry value.

In Group A, GSH concentration values showed statistically significant (s.s.) decrease (ANOVA, *p* ~ 0.04) at the surgery time interval, as compared to baseline values (admission). GSH values then returned to baseline values (Figure 1). GSH concentration values showed no s.s. differences between groups at admission (Figure 1). At surgery, patients of Group A showed non-s.s. lower (Mann-Whitney *U* test, *p* ~ 0.07) differences and at the 4^th^ and 15^th^ post-operative day time intervals non-s.s. higher (Mann-Whitney *U* test, *p* ~ 0.07) GSH values as compared to patients of Group B (Figure 1).

**Figure 2:**
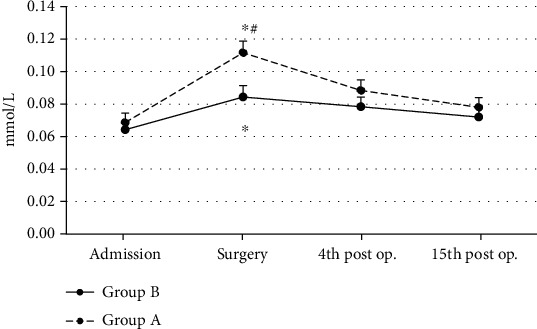
Oxidized glutathione (GSSG) mean values in both groups (*n* = 25 for each group) are shown. ^∗^Significantly different (*p* < 0.05) within groups compared with hospital entry value. ^#^Significantly different (*p* < 0.05) between groups at the same time point.

In Group A, GSSG concentration values showed s.s. increase (ANOVA, *p* ~ 0.001) at the surgery time interval, as compared to baseline values (admission). GSSG values then returned to baseline values (Figure 2). In Group B, GSSG concentration values also showed a s.s. increase (ANOVA, *p* ~ 0.02) at the surgery time interval, as compared to baseline values (admission). GSSG values then returned to baseline values (Figure 2). At surgery, patients of Group A showed s.s. higher (Mann-Whitney *U* test, *p* ~ 0.02) and at the 4^th^ postoperative day time interval non-s.s. higher (Mann-Whitney *U* test, *p* ~ 0.06) GSSG values when compared to patients of Group B (controls) (Figure 2).

**Figure 3:**
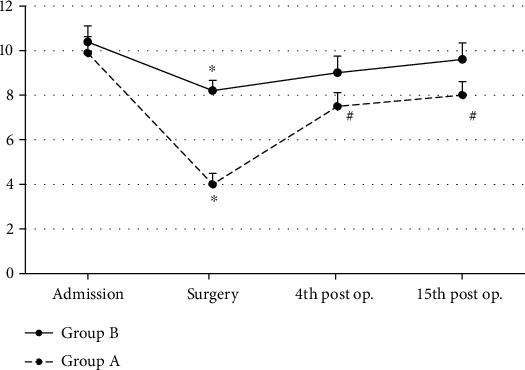
GSH/GSSG ratio mean values in both groups (*n* = 25 for each group) are shown. ^∗^Significantly different (*p* < 0.05) within groups compared with hospital entry value. ^#^Significantly different (*p* < 0.05) between groups at the same time point.

In Group A, GSH/GSSG values showed s.s. decrease (ANOVA, *p* ~ 0.001) at the surgery time interval, as compared to baseline values (admission). GSH/GSSG then remained low at the 4^th^ (ANOVA, *p* ~ 0.02) and 15^th^ (ANOVA, *p* ~ 0.04) postoperative time intervals (Figure 3). In Group B, GSH/GSSG also showed a s.s. decrease (ANOVA, *p* ~ 0.01) at the surgery time interval, as compared to baseline values (admission). GSH/GSSG then remained low at the 4^th^ (ANOVA, *p* ~ 0.04) postoperative time interval (Figure 3). Patients of Group A showed s.s. lower GSH/GSSG at surgery (Mann-Whitney *U* test, *p* ~ 0.001), at the 4^th^ (Mann-Whitney *U* test, *p* ~ 0.03) and at the 15^th^ (Mann-Whitney *U* test, *p* ~ 0.03) postoperative day time intervals as compared to patients of Group B (controls) (Figure 3).

### 3.2. Catalase

**Figure 4:**
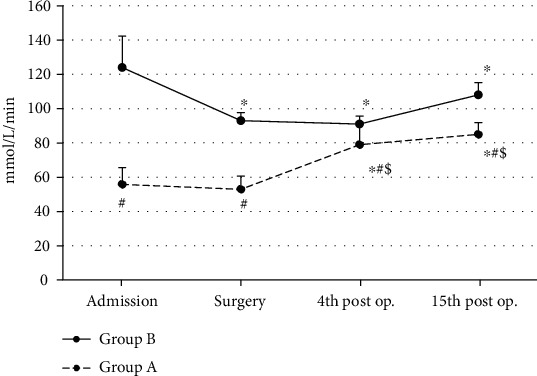
Catalase mean values in both groups (*n* = 25 for each group) are shown. ^∗^Significantly different (*p* < 0.05) within groups compared with hospital entry value. ^#^Significantly different (*p* < 0.05) between groups at the same time point. ^$^Significantly different (*p* < 0.05) between admission & 4^th^ post op.

In Group A, catalase activity showed s.s. increase at the 4^th^ (ANOVA, *p* ~ 0.01) and 15^th^ (ANOVA, *p* ~ 0.01) postoperative day time intervals as compared to admission and surgery values (baseline) (Figure 4). In Group B, catalase activity showed a s.s. decrease at the surgery (ANOVA, *p* ~ 0.01), the 4^th^ (ANOVA, *p* ~ 0.01) and the 15^th^ (ANOVA, *p* ~ 0.03) postoperative day time intervals as compared to baseline values (admission) (Figure 4). Patients of Group A showed a s.s. lower catalase activity at admission (Mann-Whitney *U* test, *p* ~ 0.001), at surgery (Mann-Whitney *U* test, *p* ~ 0.01), at the 4th (Mann-Whitney *U* test, *p* ~ 0.05), and at the 15th (Mann-Whitney *U* test, *p* ~ 0.04) postoperative day time intervals as compared to patients of Group B (controls) (Figure 4).

### 3.3. Thiobarbituric Acid Reactive Substances

**Figure 5:**
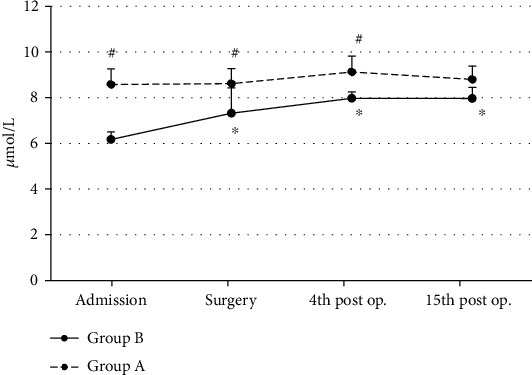
Thiobarbituric acid substance (TBARS) mean values in both groups (*n* = 25 for each group) are shown. ^∗^Significantly different (*p* < 0.05) within groups compared with hospital entry value. ^#^Significantly different (*p* < 0.05) between groups at the same time point.

In Group A, TBARS values remained unchanged during the 4 observation time intervals (Figure 5). In Group B, TBARS values showed s.s. increase at the surgery (ANOVA, *p* ~ 0.05), the 4^th^ (ANOVA, *p* ~ 0.03), and the 15^th^ (ANOVA, *p* ~ 0.03) postoperative day time intervals as compared to baseline values (admission) (Figure 5). Patients of Group A showed s.s. higher TBARS values at admission (Mann-Whitney *U* test, *p* ~ 0.01), at surgery (Mann-Whitney *U* test, *p* ~ 0.05), and at the 4th (Mann-Whitney *U* test, *p* ~ 0.05) postoperative day time intervals as compared to patients of Group B (controls) (Figure 5).

### 3.4. Protein Carbonyls

**Figure 6:**
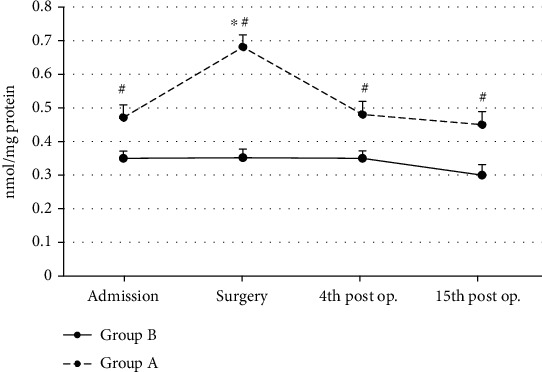
Protein carbonyl (PC) mean values in both groups (*n* = 25 for each group) are shown. ^∗^Significantly different (*p* < 0.05) within groups compared with hospital entry value. ^#^Significantly different (*p* < 0.05) between groups at the same time point.

In Group A, protein carbonyl values showed s.s. increase only at the surgery (ANOVA, *p* ~ 0.01) time interval as compared to baseline values (admission) (Figure 6). No effect was observed in Group B PC (Figure 6). Patients of Group A showed s.s. higher protein carbonyl values at admission (Mann-Whitney *U* test, *p* ~ 0.04), at surgery (Mann-Whitney *U* test, *p* ~ 0.001), at the 4th (Mann-Whitney *U* test, *p* ~ 0.04), and at the 15^th^ (Mann-Whitney *U* test, *p* ~ 0.04) postoperative day time intervals as compared to patients of Group B (controls) (Figure 6).

### 3.5. Total Antioxidant Capacity

**Figure 7:**
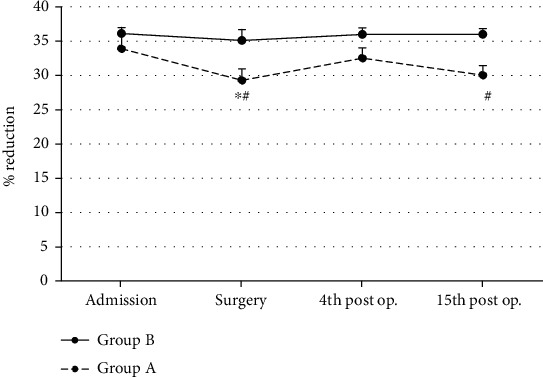
Total antioxidant capacity (TAC) mean values in both groups (*n* = 25 for each group) are shown. ^∗^Significantly different (*p* < 0.05) within groups compared with hospital entry value. ^#^Significantly different (*p* < 0.05) between groups at the same time point.

In Group A, TAC values showed s.s. decrease only at the surgery (ANOVA, *p* ~ 0.05) time interval as compared to baseline values (admission) (Figure 7). In Group B, TAC values remained unchanged during the 4 observation time intervals (Figure 7). Patients of Group A showed s.s. lower TAC values at the surgery (Mann-Whitney *U* test, *p* ~ 0.04) and at the 15^th^ (Mann-Whitney *U* test, *p* ~ 0.04) postoperative day time intervals as compared to patients of Group B (controls) (Figure 7).

## 4. Discussion

For several decades orthopaedic surgeons focused on the effective surgical management of hip fractures, perfecting either arthroplasty or internal fixation techniques without taking into account general patient health management [6, 17]. It has recently been shown that despite elaborate surgical management, hip fractures are still associated with the highest degree of morbidity and mortality of all fracture types, particularly for elderly patients [2]. Many patients never regain their independence and either suffer from major disability or die as a consequence of the hip fracture [6, 27]. A hip fracture is also a major risk for subsequent fractures [4, 6, 16, 17, 28]. Future new fractures are associated with a further increase in morbidity, mortality, and cost of management [6, 17, 28]. Our research group has also shown that patients with hip fractures present accelerated bone turnover despite early stable fracture fixation and early mobilization [17]. These patients become very fast bone losers as shown by a lumbar spine BMD reduction of 4.5% and 10.71% and a femoral neck BMD reduction of 7.4% and 9.2% at 3 and 12 months, respectively, after the hip fracture [17]. Established osteoporosis, the effect of trauma, the stress of surgery, reduced pre- and postsurgery activity, and/or a metabolic disorder such as oxidative stress may help cause the excessive rate of bone loss and may help explain the morbidity, mortality, and high incidence of new fractures in these patients. There is limited data suggesting that oxidative stress is a risk factor for hip fracture [29]. However, there is no substantiated evidence regarding the actual role of an oxidative environment on treatment, recovery, and complications of patients suffering from hip fracture. If the hypothesis that patients with hip fractures reach a metabolic status of excessive oxidative stress is correct, then we have to question what is responsible for this: the fracture, the stress of surgery, or both. Such knowledge may help explain excessive bone loss, slow functional recovery, and high rates of patient morbidity and mortality and may open new horizons for therapeutic interventions.

The results of our study show that surgery in patients with hip fractures caused significant oxidative stress with an active but insufficient first line of oxidative defense, an intensive first line reaction, a very active second line of oxidative defense, and low plasma antioxidant capacity. Surgery also worsens already present lipid- and protein-related tissue damage.

Blood redox profile can be investigated in health and disease by measuring a battery of redox biomarkers that can offer mechanistic answers after redox-altering stimuli [22, 30, 31]. Some of the most widely used redox biomarkers are GSH, GSSG, PC, CAT, TAC, and TBARS. GSH is the most abundant antioxidant thiol in the body [32]. Its role lies in detoxifying ROS, helping the body cope with oxidative stress. After intense production of ROS, GSH is converted to the oxidized form, GSSG. There is a balance between these two forms, and GSSG concentration is normally 10-fold lower than GSH concentration. Thus, increased GSSG values may be indicative of the presence of oxidative stress. The GSH/GSSG ratio is also a safe oxidative stress biomarker. A decrease in this ratio can also indicate oxidative stress. In this study, the combination of reduced GSH, increased GSSG, and reduced GSH/GSSG ratio in the hip fracture group at the second time interval shows that surgery caused ROS generation and oxidative stress in these patients recovered at a later stage. The above finding also indicates that surgery stimulated an active but insufficient first line of oxidative defense and an intensive first line reaction in hip fracture patients. Patients in the control group were affected by surgery to a lesser degree.

CAT is an antioxidant enzyme present particularly in erythrocytes and in peroxisomes. It detoxifies H_2_O_2_, an ROS that is a toxic product of both normal aerobic metabolism and abnormal ROS production [19, 23]. According to the results of the present study, the increase of CAT activity in the hip fracture group, at the third and fourth time intervals, suggests that surgery caused generation of ROS and oxidative stress. It also implies that CAT activity was very low on admission and that surgery stimulated a very active second line of oxidative defense in the hip fracture patients. Patients in the control group were affected by surgery to a lesser extent.

Regarding lipid peroxidation, hip fracture patients showed increased TBARS values throughout the observational period as compared to patients of the control group. This finding shows that lipid-related tissue damage was already present in hip fracture patients on admission. In the control group, elevated TBARS levels were observed 4 days after surgery. Similar observations were made during the healing period (five days) of fractures in rats in two previous studies [33, 34]. For the evaluation of protein and amino acid oxidation, PC were measured. ROS-mediated protein carbonylation is an important biomarker of protein oxidation, and its measurement is considered a good indicator of the extent of oxidative damage of proteins associated with various physiological and pathogenic conditions [35, 36]. In this study, hip fracture patients showed increased protein carbonyl concentration throughout the observation period with surgery further increasing the levels as compared to patients of the control group. This indicates that oxidative stress and protein-related tissue damage were already present in the hip fracture patients and that surgery further worsened them.

Finally, serum antioxidant capacity was evaluated via TAC measurement, which refers to the ability of blood components to scavenge free radicals [26]. It is an indicator of overall serum antioxidant capacity; there are a lot of molecules that account for TAC. In this study, reduced TAC levels in the hip fractures group at the second time interval suggests that surgery caused oxidative stress with low plasma antioxidant capacity which recovered at a later stage. Recovery of TAC levels is indicative of the late induction of antioxidants which have the ability to scavenge ROS. TAC levels were not affected by surgery in patients in the control group. The findings of this study suggest that patients with hip fractures who undergo surgery are more susceptible to oxidative stress with an active but insufficient first line of oxidative defense, an intensive first line reaction, a very active second line of oxidative defense, and low plasma antioxidant capacity. Surgery worsened already present lipid- and protein-related tissue damage.

The limitation of this study is the relatively small patient sample used and the relatively short observation period (two weeks). Additionally, parameters such as glucose, insulin, HOMA-IR, and lipid profile, which have an impact on the redox balance, have not been recorded. The elimination of the effect of several variables and confounding factors and the lack of drop-outs are some of the strengths of the study. Special care was taken to eliminate the effect of endocrinal and metabolic disorders, medications, and pre- and postoperative restrictions of movement by applying strict inclusion and exclusion criteria. The effect of the stress of surgery was also considered important. For this reason, we have chosen as controls patients who had undergone hip surgery for osteoarthritis for whom we have already shown that surgery has an effect on bone turnover [15].

Oxidative stress is involved in the pathology of several human diseases [37]. It is well known that increased reactive species production that causes oxidative stress is outcome of several pathologies; however, it is not known yet whether reactive species and, hence, oxidative stress are the cause of these diseases [38]. Until now it is not well understood the way that oxidative stress is related to fractures and fracture risk. In hip fractures, oxidative stress and redox regulation may be involved in the pathophysiology of the fracture and in prooxidative effects that follow the fracture [14, 39–41]. This connection may be partially explained by the elevated free radical levels observed in reduced bone density [42]. Experimental studies in cell and animal models suggest that oxidative stress is an important factor in the regulation of bone remodeling [11–13, 43]. Bone remodeling uncoupling may contribute to the regulation of prooxidant and antioxidant equilibrium in patients with osteomyelitis [44]. Previous studies have reported induction of oxidative stress during fracture healing in animal models [33, 34, 45]. Oxidative stress is involved in ankle fractures in children [46], in burst spinal fractures [47], in osteoporotic spinal fractures treated with kyphoplasty [48], and mandible fractures internally fixed with titanium alloy plates [49]. Decreased levels of antioxidant defense enzymes in femoral head fracture patients as compared to a healthy population have been reported, and it has been suggested that oxidative stress and anti-inflammatory markers like interleukins might play an important role in bone fracture pathogenesis and in bone density changes [50]. Additionally, altered plasma fatty acid status was found in patients with femoral head fractures which may affect fracture healing [51]. In an experimental fracture study in rats, antioxidant supplementation (vitamin C, vitamin E, flavonoids, and a-lipoic acid) accelerated the healing process [52]. The involvement of oxidative stress as a risk factor in the occurrence of hip fractures has been addressed in only three studies as part of the processes of age-related bone loss and slow tissue regeneration [14, 29, 53]. In general, data in the literature concerning the link between oxidative stress and bone fractures as well as fracture healing are still very scarce. Further studies are needed in order to evaluate whether manipulation of the redox balance in bone cells or the use of antioxidant therapy can improve clinical outcomes in patients with hip fractures.

In the present study, we show that patients with hip fractures have intensive oxidative stress with low total antioxidant capacity, which may explain the fact that these patients become very fast bone losers at a later stage of their life, presenting high morbidity and mortality rates. Surprisingly, three recent quality studies evaluating predictors of morbidity and mortality in hip fracture patients do not consider oxidative stress as a risk factor [54–56].

## 5. Conclusions

The management of osteoporosis and its complications, such as vertebral and hip fractures, constitutes a major socioeconomic burden. Hip fracture rates are predicted to continue to rise in the developed world because of ageing populations. Despite elaborate contemporary surgical management, hip fractures are still associated with the highest degree of morbidity, mortality, and complications. Patients with hip fractures suffer significant oxidative stress. The above findings raise the question of whether antioxidant therapy measures should be introduced in the management of hip fracture patients.

## Data Availability

The data used to support the findings of this study are available from the corresponding author upon request.

## Abbreviations

GSH: Reduced glutathione
GSSG: Oxidized glutathione
CAT: Catalase
TBARS: Thiobarbituric acid reactive substances
PC: Protein carbonyls
TAC: Total antioxidant capacity
PTH: Intact parathyroid hormone
TSH: Thyroid stimulating hormone
K2EDTA: Dipotassium ethylenediamine tetra-acetic acid
TCA: Trichloroacetic acid
H_2_O_2_: Hydrogen peroxide
DTNB: 5,5′-Dithiobis(2-nitrobenzoic acid)
DNPH: 4-Dinitrophenylhydrazine
DPPH: 1,1-Diphenyl-2-picrylhydrazyl
MDA: Malondialdehyde
BMD: Bone mineral density
ROS: Reactive oxygen species.
